# Targets and Mechanisms of Geminivirus Silencing Suppressor Protein AC2

**DOI:** 10.3389/fmicb.2021.645419

**Published:** 2021-04-09

**Authors:** Karuppannan Veluthambi, Sukumaran Sunitha

**Affiliations:** ^1^Department of Plant Biotechnology, School of Biotechnology, Madurai Kamaraj University, Madurai, India; ^2^Department of Biological Sciences, Texas Tech University, Lubbock, TX, United States

**Keywords:** geminivirus, AC2/C2, transcriptional activator, suppressor protein, silencing suppressor, biopharming, genome-editing

## Abstract

Geminiviruses are plant DNA viruses that infect a wide range of plant species and cause significant losses to economically important food and fiber crops. The single-stranded geminiviral genome encodes a small number of proteins which act in an orchestrated manner to infect the host. The fewer proteins encoded by the virus are multifunctional, a mechanism uniquely evolved by the viruses to balance the genome-constraint. The host-mediated resistance against incoming virus includes post-transcriptional gene silencing, transcriptional gene silencing, and expression of defense responsive genes and other cellular regulatory genes. The pathogenicity property of a geminiviral protein is linked to its ability to suppress the host-mediated defense mechanism. This review discusses what is currently known about the targets and mechanism of the viral suppressor AC2/AL2/transcriptional activator protein (TrAP) and explore the biotechnological applications of AC2.

## Introduction

Geminiviruses are single-stranded (ss) DNA viruses that cause major losses to a number of economically important crops throughout the world (Scholthof et al., 2011; Rojas et al., 2018). *Geminiviridae* constitutes the largest family of plant viruses with nine genera and 485 species (Zerbini et al., 2017). Geminiviruses are characterized by their small, circular, ssDNA genomes encapsidated in twinned-icosahedral particles. They are vector-transmissible and infect both monocotyledonous and dicotyledonous plants (Zerbini et al., 2017). The viral genomes are either monopartite or bipartite with circular ssDNA molecules of 2.5 to 5.5 × 10^3^ nucleotides. Bipartite geminiviruses, with DNA A and DNA B components, possess a highly conserved common region (CR) of ~200 nucleotides. An inverted repeat within the CR forms a hairpin loop, and within the loop is the conserved 9-nt sequence 5'-TAATATT^↓^AC-3'.

Geminiviruses are classified into nine genera namely *Becurtovirus*, *Begomovirus*, *Capulavirus*, *Curtovirus*, *Eragrovirus*, *Grablovirus*, *Mastrevirus*, *Topocuvirus*, and *Turncurtovirus*, based on the genome organization, host-range, and the type of insect vector which transmits the virus (Zerbini et al., 2017). Viruses in the genus *Begomovirus* have mono- or bipartite genomes while those of all other genera possess monopartite genome organization. Geminiviruses exhibit bidirectional transcription and encode 5–7 proteins that exploit and reprogram host machineries to establish infection (Hanley-Bowdoin et al., 2013; Aguilar et al., 2020). Two systems of gene nomenclature are currently in use. Both designate genes and gene products by numbers. One nomenclature denotes genes as virion-sense (V) or complementary-sense (C), whereas the other indicates genes as oriented in the rightward (R; virion-sense; clockwise) or leftward (L; complementary-sense; counter clockwise) direction of the genome map. We have used in this review, the nomenclature based on the virion-sense (V) and complementary-sense (C) strands.

**Figure 1:**
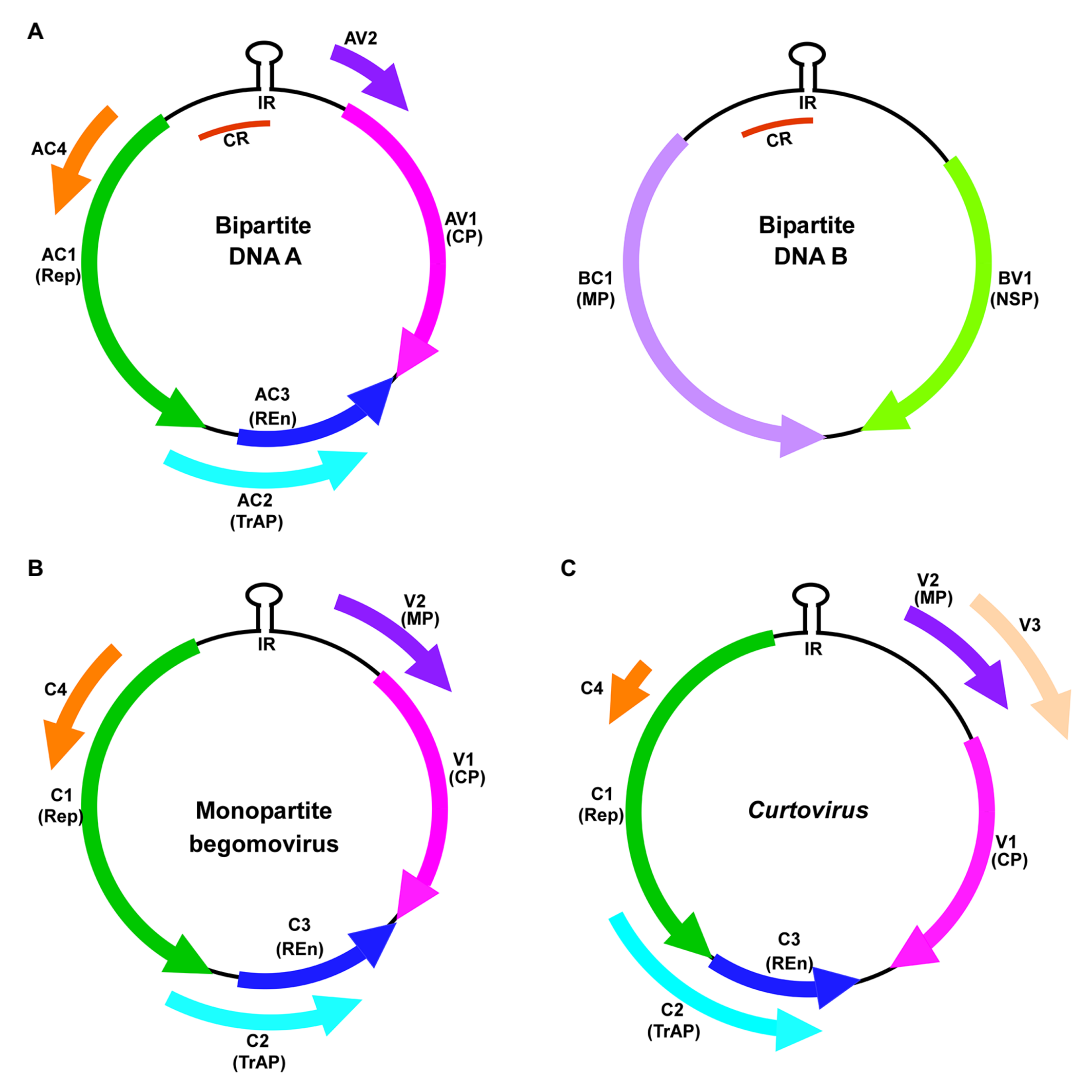
Genomic organization of **(A)** bipartite begomovirus, **(B)** monopartite begomovirus, and **(C)** curtovirus. Virion-sense (V) or complementary-sense (C) strand open reading frames (ORFs) and corresponding protein products are coded by color. CR, common region; CP, coat protein; IR, intergenic region; MP, movement protein; NSP, nuclear shuttle protein; REn, replication enhancer protein; Rep, replication-associated protein; and TrAP, transcriptional activator protein.

*Begomovirus* represents the largest and the best-studied geminivirus genus. The bipartite genomes of begomoviruses are designated as DNA A and DNA B (Figure 1A). DNA A encodes five or six open reading frames (ORFs) and DNA B encodes two ORFs. The virion-sense ORFs AV1 and AV2 of DNA A encode coat protein (CP) and pre-coat protein, respectively (Padidam et al., 1996). The complementary-sense strand ORFs of DNA A encode the replication-associated protein (AC1/Rep), the transcriptional activator protein (AC2/TrAP), the replication enhancer protein (AC3/REn), and AC4 (Hanley-Bowdoin et al., 2000). DNA B ORFs encode the nuclear-shuttle protein (BV1/NSP) and the movement protein (BC1/MP; Schaffer et al., 1995).

Monopartite begomoviruses lack the DNA B component (Kheyr-Pour et al., 1991; Navot et al., 1991). The single, circular ssDNA genome encodes six ORFs, two in the virion-sense (V1 and V2) and four in the complementary-sense (C1, C2, C3, and C4) strands (Figure 1B). Some monopartite begomoviruses do not cause typical disease symptoms in infected plants when acting alone. They require an ssDNA satellite molecule (betasatellite/alphasatellite/deltasatellites/defective satellites) to cause symptomatic disease and viral DNA accumulation (Saunders et al., 2000; Fiallo-Olivé et al., 2012, 2016).

Curtovirus genome organization is like that of monopartite begomoviruses (Figure 1C). The virion-sense strand of curtoviruses is more complex with three ORFS V1, V2, and V3. V1 encodes the coat protein and V3 encodes the movement protein. V2/Reg is a unique curtovirus protein that regulates relative ssDNA and dsDNA levels and hence the name “Reg” (Hormuzdi and Bisaro, 1993). The complementary-sense strand encodes four genes namely Rep/C1, C2, REn/C3, and C4 (Stanley and Latham, 1992; Stanley et al., 1992). Rep/C1 (Luna and Lozano-Durán, 2020) and Ren/C3 are highly conserved between curtovirus and begomovirus. Curtovirus REn was shown to functionally complement the REn mutation in a bipartite begomovirus (Hormuzdi and Bisaro, 1995). However, curtovirus C2, and the positional homolog of begomovirus AC2, display only partial sequence and functional homology between them (Luna and Lozano-Durán, 2020).

**Figure 2:**
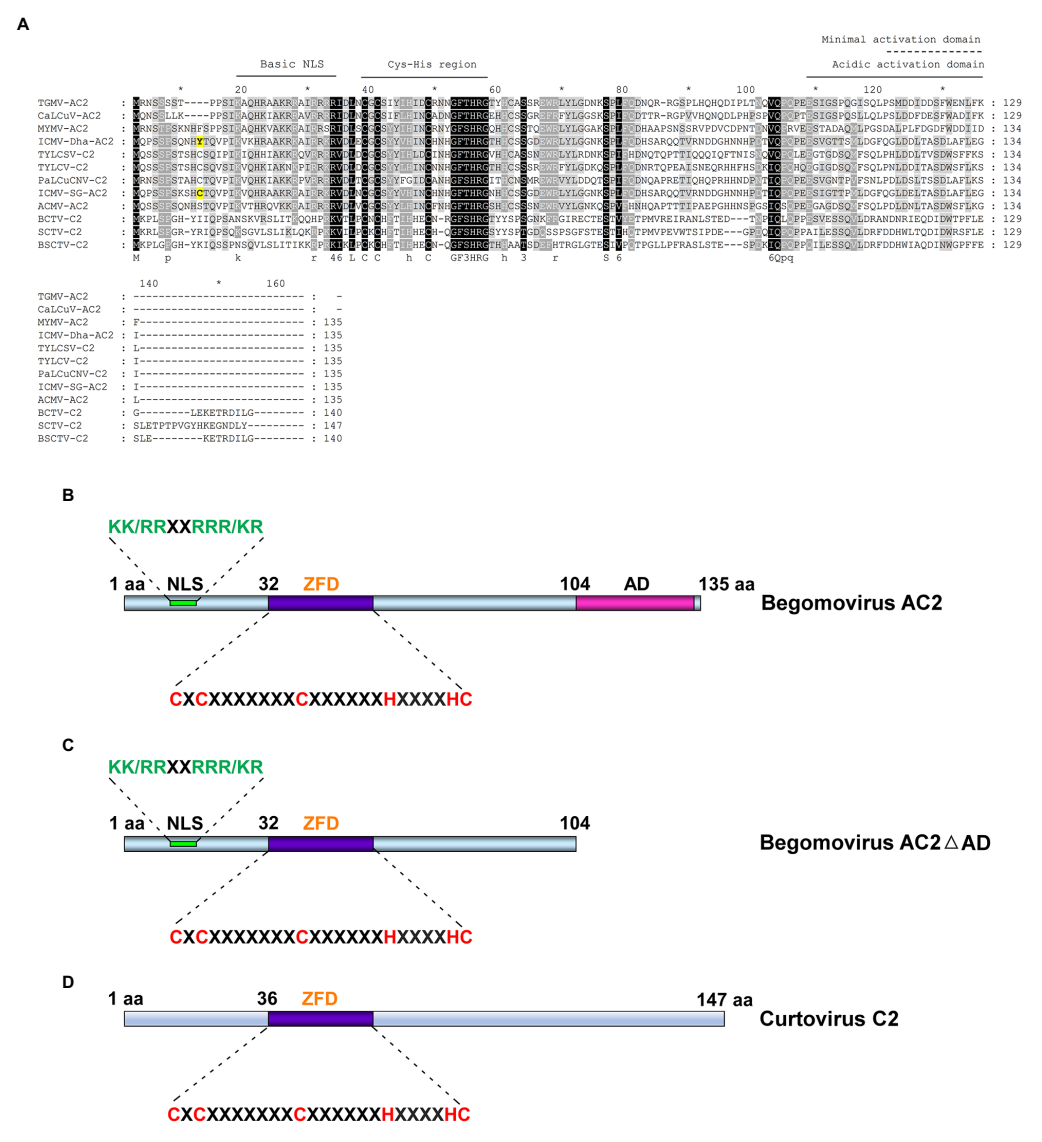
Alignment of AC2 of bipartite, C2 proteins of monopartite begomovirus, and C2 of curtovirus. **(A)** Clustal alignment of AC2/C2 amino sequences of *Tomato golden mosaic virus* (TGMV; acc. no. NC_001507); *Mungbean yellow mosaic virus* (MYMV; acc. no. AJ132575); *African cassava mosaic virus* (ACMV; acc. no. NC_001467); *Indian cassava mosaic virus*-Singapore (ICMV-SG; acc. no. JX518289); *Indian cassava mosaic virus*-Dharwad (ICMV-Dha; acc. no. GQ924760); *Cabbage leaf curl virus* (CaLCuV; acc. no. NC_00386); *Tomato yellow leaf curl Sardinia virus* (TYLCSV; acc. no. L27708); *Tomato yellow leaf curl virus* (TYLCV; acc. no. AM282874); *Papaya leaf curl China virus* (PaLCuCNV; acc. no. FN256260); *Beet curly top virus* (BCTV; acc. no. AF379637); *Beet severe curly top virus* (BSCTV; acc. no. U02311); and *Spinach curly top virus* (SCTV; Acc. No. AY548948). The conserved N-terminal basic domain, the Cys-His residues in the middle and the C-terminal acidic activation domain are marked. The Tyr residue in ICMV-Dha strain and Cys residue in pathogenic ICMV-SG strain are highlighted in yellow. **(B)** Schematic representation of begomovirus AC2 involved in activation domain-dependent silencing suppression. **(C)** Schematic representation of begomovirus AC2 with deletion in activation domain (AC2ΔAD) involved in activation domain-independent silencing suppression. **(D)** Schematic representation of curtovirus C2 involved in activation domain-independent silencing suppression. NLS, nuclear localization signal; ZFD, zinc-finger like domain; and AD, activation domain.

Although the position of AC2/C2 ORFs is conserved in most geminivirus genera, the functions of AC2/C2 are known only in curtovirus and begomovirus. AC2, a 15 kDa multifunctional protein, is also known as C2, L2, AL2, or transcriptional activator protein (TrAP). AC2 is a delayed early gene product which transactivates late viral genes, *CP*, and *NSP* (Sunter and Bisaro, 1991, 1992; Haley et al., 1992; Shivaprasad et al., 2005), suppresses host defense mechanism (Bisaro, 2006; Raja et al., 2010), and acts as a symptom determinant (Hao et al., 2003; Rajeswaran et al., 2007; Siddiqui et al., 2008). *C2*, a positional homolog of *AC2* in monopartite begomoviruses, is highly similar to AC2 in sequence and function (Figure 2A; Noris et al., 1996; van Wezel et al., 2001, 2002, 2003; Dong et al., 2003; Gopal et al., 2007). The C2 protein which is encoded by the curtoviruses *Beet curly top virus* (BCTV), *Beet severe curly top virus* (BSCTV), and *Spinach curly top virus* (SCTV) exhibits very little sequence similarity when compared to begomovirus AC2/C2 except for the conserved Cys-His residues in the middle (Figures 2A,D; Luna and Lozano-Durán, 2020). AC2 has three highly conserved functional domains: (1) N-terminal basic domain with a bipartite nuclear localization signal (NLS), which consists of four consecutive arginine residues (Figures 2A–C; Dong et al., 2003; Trinks et al., 2005). (2) A conserved zinc finger-like domain comprising the conserved cysteine and histidine residues (CCHC) which is present in the middle. (3) The C-terminal which possesses an acidic transactivation domain (Figures 2A,B; Trinks et al., 2005). This review focuses on the host targets and versatile mechanisms deployed by the geminiviral silencing suppressor protein AC2/C2 to counter the plant defense.

## Plant Defense and Viral Counter-Defense Mechanisms

**Figure 3:**
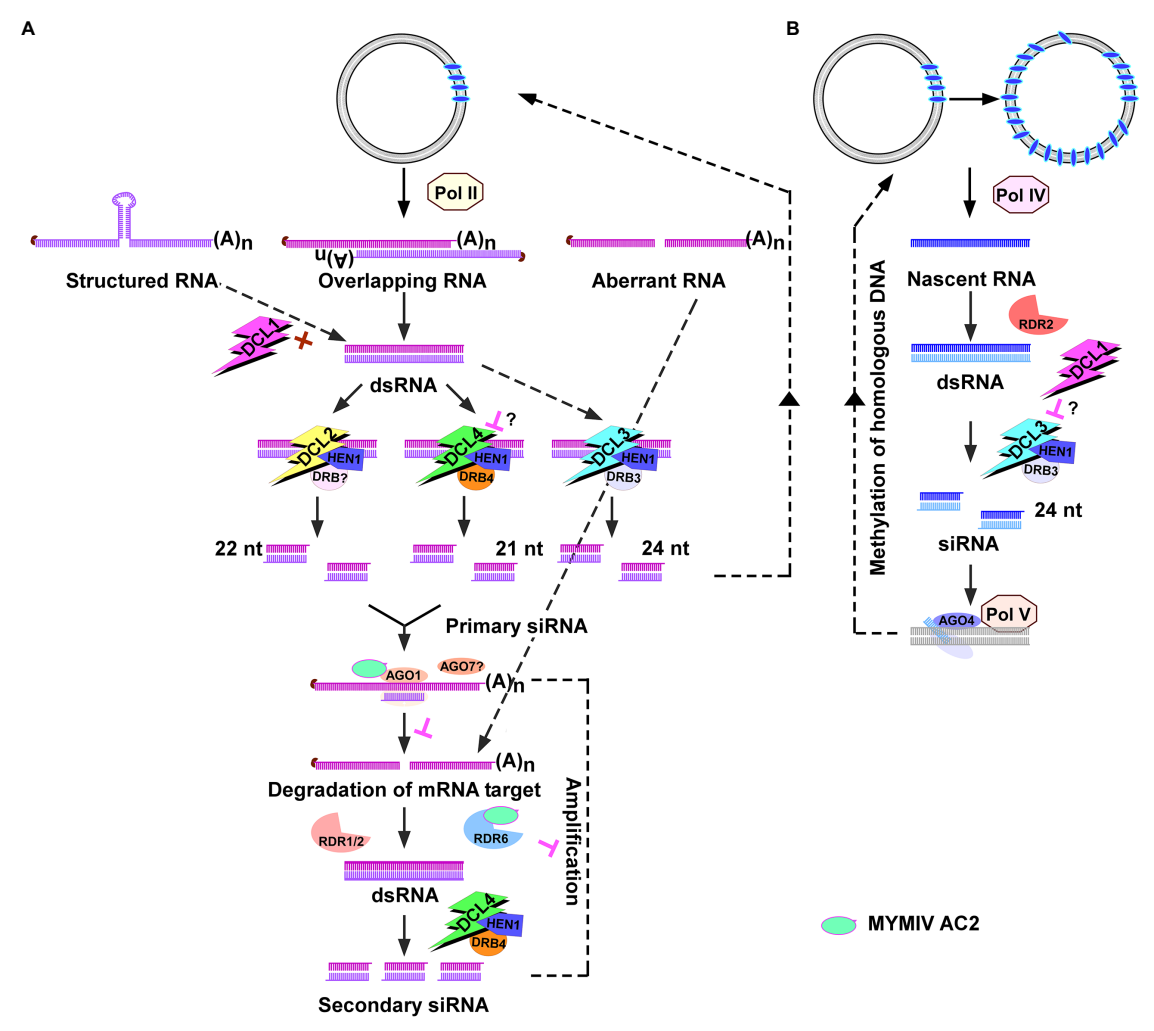
Geminiviral siRNA pathways. **(A)** Post-transcriptional gene silencing (PTGS) pathway. The host RNA polymerase transcribes the replicative form of virus into structured/overlapping/aberrant transcripts which are processed into 21 nt siRNA by DICER-LIKE4/DCL4. DCL2 generates 22 nt siRNAs in the absence of DCL4. DCL1 acts as a positive regulator (+) by facilitating dsRNA access to other DCLs. The role of DCL1 as a negative regulator (┴) of DCL4 and DCL3 in geminiviral siRNA pathway is not clear (?). The 21 nt siRNAs-Argonaute1 (AGO1) target viral transcripts for slicing by the endo-ribonuclease activity of AGO1. The role of AGO7 in geminiviral defense is not clear (?). The sliced mRNA transcript serves as a template for RNA-DEPENDENT RNA POLYMERASE1/2/6 (RDR1, -2, -6) to convert it into dsRNA, which thereafter generates secondary siRNAs to amplify the host defense response. Suppression of PTGS by binding AGO1 and RDR6 by *Mungbean yellow mosaic India virus* (MYMIV) AC2 is denoted. **(B)** Transcriptional gene silencing (TGS) pathway. The Pol II transcribed dsRNA is processed by DCL3 (when DCL2 and DCL4 are saturated) into 24 nt siRNAs which cause *de novo* methylation of viral genome. The methylated viral genome is transcribed by Pol IV to produce a nascent transcript, which is converted to dsRNA by RNA-DEPENDENT RNA POLYMERASE2/RDR2. dsRNA is processed by DCL3 into 24 nt siRNAs. The complex of 24 nt siRNA-AGO4 targets the Pol V-generated scaffold transcript to mediate RNA-directed DNA methylation. DRB, double-stranded RNA binding proteins and HEN1, HUA ENHANCER1 methyltransferase.

RNA silencing is a very effective antiviral defense mechanism. RNA silencing has evolved as the first line of defense against invading nucleic acids including viruses, transposons, transgenes, and repetitive sequences (Baulcombe, 2004; Voinnet, 2005; Shabalina and Koonin, 2008). In addition to structured ssRNA viral genomes, DNA viral transcripts which are structured and overlapping transcripts also act as precursors for viral siRNA pathway (Blevins et al., 2011; Aregger et al., 2012). The dsRNA is processed into 21 nt siRNAs by DICER-LIKE4/DCL4 protein (Figure 3A; Akbergenov et al., 2006; Blevins et al., 2006). DCL2 expression stimulates transitivity and secondary siRNA production and increases silencing efficiency in the absence of DCL4 (Parent et al., 2015). When DCL2 and DCL4 are present together, abundant RNAs from viruses and transgenes are processed hierarchically first by DCL4 which has high affinity and processivity to restrict off-target silencing caused by the secondary siRNAs generated by the transitivity-prone DCL2 (Parent et al., 2015). DCL1-mediated processing of geminiviral dsRNA into 21 nt siRNA is inefficient (Blevins et al., 2006); however, it acts as a positive regulator by making viral dsRNAs available to other DCLs to be processed into siRNAs (Blevins et al., 2006; Csorba et al., 2015). DCL1 also acts as a negative regulator of viral silencing by downregulating DCL4 and DCL3 (Qu et al., 2008). The 21 nt siRNA generated by DCL4 cleaves the target viral transcript in association with ARGONAUTE (AGO) proteins. AGO2 acts as a second layer of defense when AGO1 is suppressed in *Arabidopsis* (Harvey et al., 2011). AGO7 acts in coordination with AGO1 for viral clearance (Qu et al., 2008) but preferentially targets less structured viral RNA. The role of AGO7 in geminiviral defense is not clear.

The AGO-siRNA sliced target transcript serves as a template for RNA-DEPENDENT RNA POLYMERASE1/RDR1, RDR2, and RDR6 to convert it into dsRNA, which thereafter generates secondary siRNAs in RNA viruses (Garcia-Ruiz et al., 2010). Interestingly, geminiviral mRNAs appear to be poor templates for RDR-dependent secondary siRNA biogenesis. Aregger et al. (2012) showed that the viral siRNAs which accumulate in *Cabbage leaf curl virus* (CaLCuV)-infected *Arabidopsis* are RDR1/2/6-independent primary siRNAs. Interestingly, RDR6 was shown as a target of *Mungbean yellow mosaic India virus* (MYMIV) AC2 (Kumar et al., 2015). Although DCL4 has been implicated in the generation of 21 nt siRNAs, DCL2 and DCL3 appear to work in concert to generate the antiviral response (Figures 3A,B). Abundance of 21, 22, and 24 nt siRNAs is observed in cassava and *Nicotiana benthamiana* infected with *African cassava mosaic virus* (ACMV; Akbergenov et al., 2006). Prevalence of 21–22 nt siRNAs was observed in tomato infected with *Tomato yellow leaf curl Sardinia virus* (TYLCSV; Miozzi et al., 2013) and preferential accumulation of 22 nt siRNAs was observed in tomato and *N. benthamiana* infected with *Tomato yellow leaf curl China virus* (TYLCCNV; Yang et al., 2011). Interestingly, 24 nt siRNAs generated by DCL3 (Figure 3B) are the most abundant siRNAs in *Arabidopsis* infected with CaLCuV (Blevins et al., 2006).

RNA-directed DNA methylation (RdDM) of cytosine residues and histone H3 lysine 9 dimethylation (H3K9me2) of chromatin are hallmarks of epigenetic defense mechanism evolved by the plants against invading DNAs including geminiviruses and transposons. The equilibrium between repressed and active viral chromatin determines the outcome of infection and symptom remission. Host recovery is tightly associated with the equilibrium favoring repressed state (Ceniceros-Ojeda et al., 2016; Coursey et al., 2018). The canonical RdDM machinery includes RNA DEPENDENT RDR2, DCL3, AGO4, and two plant-specific RNA polymerases, Pol IV and Pol V (Zhang et al., 2018). Pol IV transcribes heterochromatic regions to produce a nascent transcript (Herr et al., 2005; Daxinger et al., 2009), which is recruited to the cajal bodies in the nucleolus, where they are converted into dsRNA by RDR2 (Li et al., 2006). The resultant dsRNA is processed by DCL3 into 24 nt siRNAs, which are methylated by HEN1 and used by AGO4 to base pair with the Pol V-generated scaffold transcript (Wierzbicki et al., 2008) to mediate RNA-directed DNA methylation (Figure 3B). Scaffold transcripts which originate from intergenic non-coding sequences are required for silencing adjacent siRNA-generating loci (Wierzbicki et al., 2009). AGO4 recruits chromatin modifying methyltransferase (MTase) and also slices the scaffold transcript which serves as a template for RDR2-mediated dsRNA production and amplification of 24 nt siRNA. Methylation of the invading geminiviral genome is mediated by 24 nt siRNAs (Raja et al., 2008; Buchmann et al., 2009). Interestingly, Pol IV and Pol V were shown to be not essential for *de novo* methylation of geminiviral genome (Jackel et al., 2016). *Arabidopsis* mutants *pol IV* and *pol V* reduced accumulation of all classes of virus-derived siRNAs suggesting that they are not so essential in viral siRNA biogenesis including 24 nt siRNA biogenesis. While Pol IV and Pol V were not essential in establishing cytosine methylation in the viral genome, they were found to be critical for maintenance and amplification of methylation. Incidentally, the mechanism involving Pol II-RDR6-mediated methylation of retrotransposon (Marí-Ordóñez et al., 2013) was evoked to explain the *de novo* methylation of geminiviral genome (Jackel et al., 2016; Figure 3B). A study by Marí-Ordóñez et al. (2013) showed that EVADE (EVD), a retrotransposon, generates high levels of Pol II-RDR6 dependent dsRNA upon proliferation which could eventually quench the DCL2 and DCL4 dicers involved in 21 and 22 nt siRNA biogenesis. Thus, the Pol II-RDR6 dependent dsRNA are processed by DCL3 into 24 nt siRNAs from transcribed regions of the retrotransposon. The key role of Pol IV and Pol V in establishing chromatin methylation and in enabling recovery emphasized the importance of these enzymes in mounting antiviral defense through methylation.

The presence of a robust viral counter defense mechanism is underscored by the ubiquitous presence of one or more silencing suppressor proteins in the armor of a virus. The arms race between silencing and silencing suppression results in resistance or susceptibility to the pathogen. Geminiviruses encode several proteins namely AC2/C2, AC4/C4, AV2/V2, βC1, and Rep that suppress RNA silencing by targeting various components of the post-transcriptional gene silencing (PTGS) machinery, transcriptional gene silencing (TGS) machinery, and cellular regulatory genes (reviewed in Hanley-Bowdoin et al., 2013; Kumar, 2019; Rishishwar and Dasgupta, 2019; Yang et al., 2019; Guerrero et al., 2020). Of all the known geminiviral suppressor proteins, AC2 is the most well-studied and is known to target multiple plant genes and proteins. In this review, we have taken a comprehensive approach to document all known targets of the geminiviral suppressor protein AC2 and the interconnecting and/or unique mechanisms evolved by the viruses to counter the plant defense mechanism.

## AC2-Mediated Suppression of Host Defense Mechanism

AC2 of begomoviruses and C2 of curtoviruses act as suppressors of silencing through two broadly classified mechanisms: (i) activation domain-dependent silencing suppression- begomovirus AC2 with a C-terminal acidic activation domain (Figure 2B) is involved in this mechanism and (ii) activation domain-independent silencing suppression, which is manifested by curtovirus C2 which lacks the activation domain (Figure 2D) and certain begomovirus AC2 proteins with deletions of the activation domain (Figure 2C). AC2/C2 also interacts with and inactivates many cellular regulatory proteins to circumvent the innate defense mechanism which is independent of siRNA-mediated silencing (Guerrero et al., 2020).

### Activation Domain-Dependent Silencing Suppression

Suppression activity of AC2 was first demonstrated by Voinnet et al. (1999) in the *N. benthamiana* line 16c, in which the previously established silencing of *gfp* was reverted by the expression of the ACMV *AC2* in a potato virus X (PVX) vector. Similar studies with C2 of *Tomato yellow leaf curl virus*-China (TYLCV-C), a monopartite begomovirus, revealed that the suppression of silencing was associated with the presence of an intact zinc finger-like motif (van Wezel et al., 2002) and an NLS (Dong et al., 2003). *East African cassava mosaic Cameroon virus* (EACMCV) and *Indian cassava mosaic virus* (ICMV) are known to suppress silencing (Vanitharani et al., 2004). Although the mechanism of suppression is not clear, transient AC2 expression enabled ~8-fold increase in synergistic mixed infection with recovery type viruses ACMV and *Sri Lankan cassava mosaic virus* (SLCMV), which exhibit recovery from symptoms 2 to 3 weeks after infection (Vanitharani et al., 2004). The activation domain-dependent silencing suppression is also known as “transcription-dependent silencing suppression” (Bisaro, 2006). Trinks et al. (2005) demonstrated the requirement of the intact activation domain of *Mungbean yellow mosaic virus* (MYMV) AC2 in addition to the NLS and Zn-finger motifs to suppress silencing. The need for NLS, Zn-finger motif, and activation domain of AC2 for efficient silencing suppression suggested that MYMV and ACMV AC2 and TYLCV-C C2 regulate silencing suppression in the host cell nucleus and are dependent on DNA interaction and transcriptional activation.

#### Suppression of PTGS by Transactivation of Host Suppressor *WEL1*

**Figure 4:**
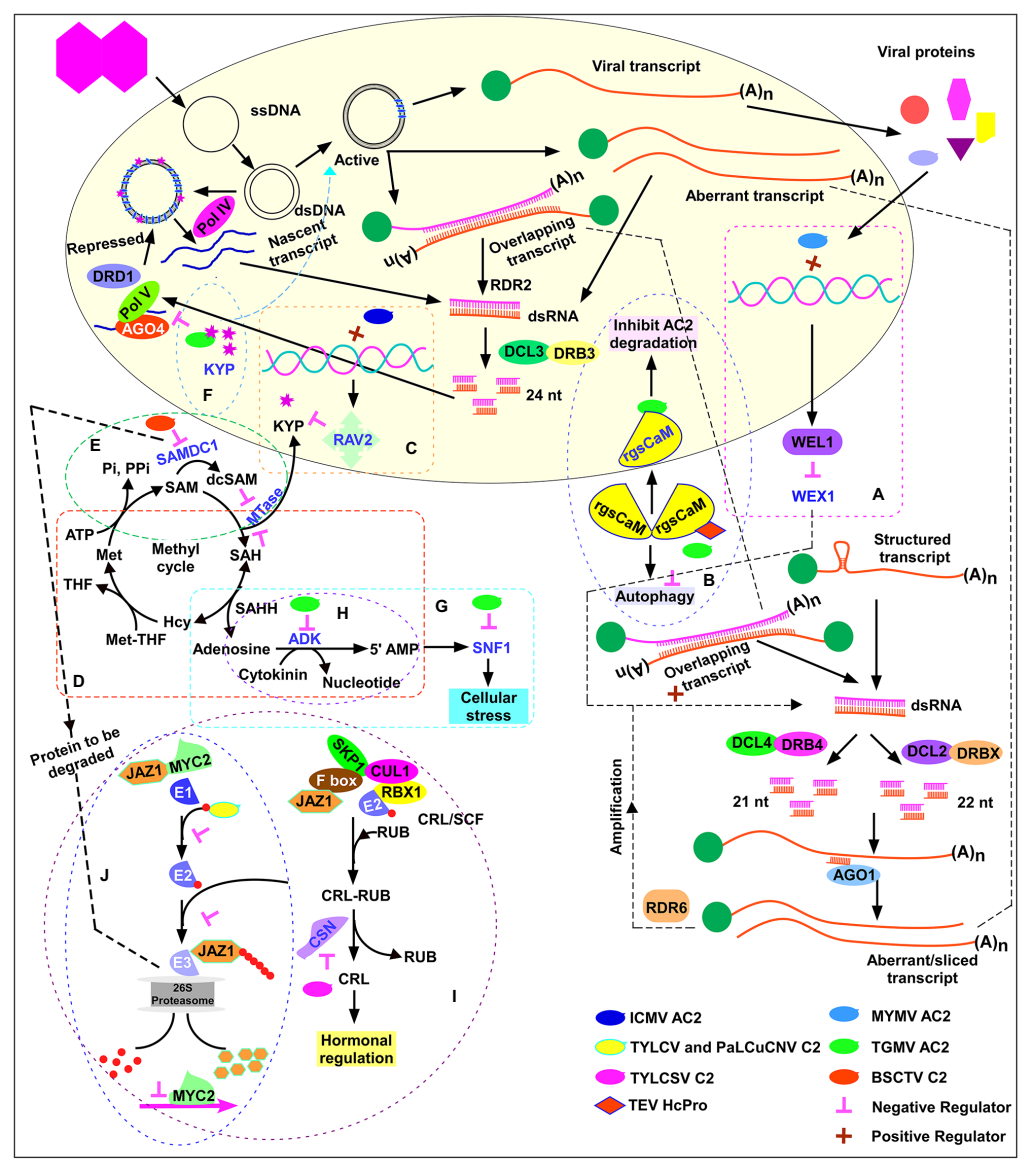
AC2-mediated suppression of host defense mechanism. Activation domain-dependent mechanism: suppression of PTGS by activating the host silencing suppressors **(A)** Werner’s exonuclease-like 1 (*WEL1*) by MYMV AC2, **(B)** calmodulin-like protein by TGMV AC2, and **(C)** RELATED TO ABI3 and VP1 (RAV2), transcription repression and downregulation of H3K9 histone methyltransferase KRYPTONITE (KYP) by *Indian cassava mosaic virus* (ICMV) AC2. Activation domain-independent mechanism: **(D)** The methyl cycle and suppression of PTGS/TGS by inactivating adenosine kinase by TGMV AC2 and BCTV C2. **(E)** Suppression of PTGS by stabilizing S-adenosyl methionine decarboxylase1 by BSCTV C2. **(F)** Suppression of TGS by inhibiting KYP enzymatic activity by TGMV and CaLCuV AC2 binding. **(G)** Suppression mediated by inactivation of SNF1 kinase by TGMV AC2 and BCTV C2. **(H)** Suppression mediated by elevation of cellular cytokinin levels by TGMV AC2 and C2 of SCTV. **(I)** Suppression mediated by inhibition of jasmonate signaling pathway by C2 of TYLCSV. **(J)** Suppression mediated by competitive binding of C2 of TYLCV and PaLCuCNV to ubiquitin.

Transient expression of MYMV and ACMV *AC2* in *Arabidopsis* protoplasts upregulated the expression of 30 plant genes. One such gene was Werner’s exonuclease-like 1 (*WEL1*), a homolog of Werner Syndrome-like exonuclease (*WEX*). WEX, an RNase D exonuclease-like protein acts as a positive regulator of post-transcriptional gene silencing (PTGS; Glazov et al., 2003). WEL1 does not have the complete “DEDDY” signature conserved in WEX. Hence, AC2-mediated upregulation of *WEL1* is likely to exert a dominant negative effect on WEX function (Figure 4A; Trinks et al., 2005). Thereby, a novel mechanism of AC2-mediated induction of host silencing suppressors was proposed by Trinks et al. (2005). The authors showed induction of *WEL1* and five other genes by MYMV and ACMV AC2 in *Arabidopsis* and *Nicotiana plumbaginifolia* protoplasts. However, upregulation of *WEL1* upon ACMV or MYMV viral infection or an increase in viral load upon transgenic over-expression of *WEL1* has not been demonstrated to date; thus, the role of *WEL1* as a host silencing suppressor remains to be confirmed. Similarly, the authors did not evaluate whether silencing of *WEL1* abolished AC2-mediated suppression in *N. benthamiana* 16c line. Interestingly, Trinks et al. (2005) demonstrated that mutations of all three functional domains (NLS1-, ZF-, and AD-) abolished transactivation as well as silencing suppression property of AC2. MYMV AC2 being a small, multifunctional protein, the loss of silencing suppression of AC2 mutants by impaired folding or loss of interaction with host factors cannot be discounted.

#### Suppression of PTGS by Transactivation of Calmodulin-Like Protein

*Tomato golden mosaic virus* (TGMV) AC2 induces a calmodulin-like protein Nb-rgsCaM (Chung et al., 2014). rgsCaM over-expression leads to an increase in viral DNA load. rgsCaM was shown to be induced by HcPro, a silencing suppressor of *Tobacco etch virus* (TEV; Anandalakshmi et al., 1998) and over-expression of rgsCaM was shown to reverse PTGS (Anandalakshmi et al., 2000), suggesting the role of rgsCaM as an endogenous silencing suppressor. rgsCaM prevents TEV HcPro and Cucumber mosaic virus suppressor 2b from binding to dsRNAs/siRNAs and reduce the suppressor protein stability by autophagy, resulting in a more potent RNAi defense against viral infection. rgsCaM over-expressing lines were less susceptible to the virus (Nakahara et al., 2012). In contrast, rgsCaM over-expression resulted in increased susceptibility to TGMV and CaLCuV (Chung et al., 2014) likely because TGMV and CaLCuV are DNA viruses, whereas the viruses studied by Nakahara et al. (2012) were RNA viruses. TGMV AC2 does not bind dsRNAs/siRNAs unlike RNA viral suppressors HcPro, P19, and 2b. rgsCaM self-interaction was observed in cytoplasm while interaction with TGMV AC2 sequestered rgsCaM to the nucleus (Figure 4B). It was speculated that AC2-mediated localization of rgsCaM to the nucleus is the likely mechanism evolved by TGMV to evade degradation of AC2 by autophagy and thereby effectively suppress the plant defense mechanism. The cajal bodies in the nucleolus are the sites of 24 nt siRNA biogenesis (Pontes and Pikaard, 2008). Chung et al. (2014) speculated that nuclear localization of rgsCaM by TGMV AC2 might interfere with the overall host siRNA biogenesis and make the plants more susceptible to the virus. More studies are needed in future to confirm this hypothesis. TYLCCNV βC1 was shown to act as suppressor of PTGS by upregulating rgsCaM (Li et al., 2014). A recent study showed that TYLCCNV βC1-upregulated rgsCaM interacts with Suppressor of Gene Silencing 3 (SGS3), a cofactor of RDR6 and induces autophagic degradation of it and thereby suppresses PTGS (Li et al., 2017).

#### Suppression of TGS by Transactivation of Host Repressor

*Indian cassava mosaic virus*-Singapore (ICMV-SG) displayed higher pathogenicity in comparison to ICMV-Dharwad (ICMV-Dha). A single point mutation that changes Tyr to Cys in ICMV-Dha AC2 (ICMVDha^Y11C^) significantly increased pathogenicity (Figure 2A; Sun et al., 2015). Increased ICMV-SG infection was associated with increased repression of H3K9 histone MTase KRYPTONITE (NbKYP), a key enzyme for maintenance of chromatin methylation. Downregulation of KYP was directly correlated with an increase in RELATED TO ABI3 and VP1 (RAV2), a transcription repressor. RAV2 is known to regulate RNA silencing and to get upregulated by suppressor proteins of potyvirus HcPro and cucomovirus P38 in *Arabidopsis* (Endres et al., 2010). Transient expression of ICMV-SG, ICMV-Dharwad (ICMV-Dha), and the ICMV-Dha^Y11C^AC2 mutant in *N. benthamiana* 16c-TGS reversed the TGS of the 16c line as evidenced by reactivation of green fluorescence (Sun et al., 2015). Silencing of *NbRAV2* significantly reduced the viral titer, thus indicating a novel mechanism of silencing suppression. Activation of the putative transcription repressor *NbRAV2* by ICMV-AC2 interferes with TGS by suppressing the expression of KYP (Figure 4C). It is not known whether the RAV2 repressor protein directly binds to the *KYP* promoter sequence to downregulate its expression. A RAV2-dependent upregulation of *Arabidopsis* FIERY1 (FRY1) and CML38 (rgsCaM homolog) was observed in TuMV HC-Pro transgenic lines (Endres et al., 2010). It will be useful to study whether ICMV-SG induced RAV2 protein can induce rgsCaM in *N. benthamiana*.

### Activation Domain-Independent Silencing Suppression

Reports on AC2-mediated silencing suppression in TGMV suggest that not all viruses require C-terminal activation domain in AC2 for mediating silencing suppression. The activation domain-independent silencing suppression observed in TGMV is also referred as “transcription-independent silencing suppression” (Bisaro, 2006). The unique mechanism evolved by the viruses encoding activation domain-independent silencing suppressors is by interfering with the methyl cycle. S-adenosyl methionine (SAM) is the methyl donor for most transmethylation reactions and is an essential MTase co-factor. An increase in the accumulation of SAM analogs would competitively inhibit MTase and prevent methylation and associated silencing. Activation domain-independent silencing suppressors interfere with the methyl cycle and increase the cellular levels of SAM analogs which compete with SAM. In addition to methyl cycle interference, TGMV and CaLCuV AC2 directly target and inhibit the H3K9me2 histone MTase Su(var)3-9 homolog 4/Kryptonite (SUVH4/KYP), an enzyme critical for histone methylation.

#### Suppression of PTGS by Inactivating Adenosine Kinase

TGMV AC2 with activation domain deletion (AC2-∆AD; Figure 2C) and BCTV C2 (Figure 2D) interact with adenosine kinase (ADK) and inhibit the synthesis of 5'-AMP from adenosine and ATP (Figure 4D; Wang et al., 2003). C2 of BCTV and C2 of SCTV, both curtoviruses, lack the transcriptional activation domain which is present in AC2/C2 of begomoviruses (Sunter et al., 1994; Baliji et al., 2007). Although AC2 self-interacts and moves into the nucleus for transcriptional activation, AC2:ADK and C2:ADK complexes form in the cytoplasm (Yang et al., 2007). This emphasizes the dispensability of the activation domain for ADK interaction. AC2/C2 expression inhibited ADK activity in *Escherichia coli*, yeast, and transgenic plants (Wang et al., 2003). ADK plays a key role in the methyl cycle and in SAM-dependent MTase activity. MTase catalyzes methyl group transfer from SAM to a methyl acceptor converting SAM to S-adenosyl-homocysteine (SAH). S-adenosyl-homocysteine hydrolase (SAHH) hydrolyzes SAH to homocysteine (Hcy) and adenosine (Figure 4D). SAHH catalyzed reaction is reversible, and the removal of adenosine is essential to tilt the equilibrium of reaction toward hydrolysis of SAH, which otherwise lies strongly toward synthesis of SAH. SAH is also a competitive inhibitor of MTase. Phosphorylation of adenosine to 5'-AMP by ADK prevents resynthesis of SAH and promotes flux through the methyl cycle to regenerate SAM. Removal of adenosine by phosphorylation facilitates methionine (Met) synthesis, wherein methionine synthase catalyzes the transfer of a methyl group from methylated folic acid [methyltetrahydrofolate (MTHF)] to homocysteine. Addition of adenosine to the sulfur group of methionine regenerates SAM. Thus, the removal of SAH is critical as it can strongly compete with SAM and inhibit MTase (Figure 4D). ADK-deficient plants displayed defects in silencing, thus implying an indirect role for ADK in regulating methyl cycle and silencing.

Wild-type *AC2* and *AC2*-∆AD of TGMV and *C2* of BCTV suppressed PTGS which was directed against *gfp* in the *N. benthamiana* 16c line in an activation domain-independent manner (Wang et al., 2005). The local suppression caused by AC2 could be mimicked by using an invert repeat of ADK or by addition of an adenosine homolog (A-134974) that inhibited ADK activity (Wang et al., 2005). Methylation of the coding region of a gene is a hallmark of PTGS (Ingelbrecht et al., 1994; English et al., 1996). Thus, AC2-mediated inactivation of ADK is likely to have caused suppression of PTGS by its interference in the methylation of coding region (Raja et al., 2010).

#### Suppression of PTGS by Stabilizing S-Adenosyl Methionine Decarboxylase1

BSCTV C2 interaction with S-adenosyl methionine decarboxylase1 (SAMDC1) and resultant suppression of *de novo* DNA methylation were observed in *Arabidopsis* (Zhang et al., 2011). SAMDC1 is a key enzyme in the conversion of SAM to decarboxylate-S-adenosyl methionine (dcSAM). dcSAM acts as an aminopropyl donor for the biosynthesis of spermidine and spermine. dcSAM also competes with SAM and acts as a competitive inhibitor of MTase (Figure 4E). Hence, SAM/dcSAM balance is a key determinant of transmethylation. SAMDC1 possesses a conserved PEST (proline, glutamine, serine, and threonine) sequence that is associated with proteins with rapid turnover rates. BSCTV C2 interacts with SAMDC1 in the PEST region and stabilizes the enzyme by attenuating 26S proteasome-mediated degradation (Figure 4E). The resultant increase in dcSAM/SAM ratio affects the host *de novo* methylation. Infection of wild-type plants with BSCTV C2^−^ mutant strain (engineered by introducing a stop codon in the C2 ORF) or infection of SAMDC1 mutant plant with wild-type BSCTV strain resulted in enhanced DNA methylation, reduced viral titer and reduced susceptibility confirming the key role of C2 and SAMDC1 in regulating host DNA methylation. Agroinfiltration of BSCTV *C2* and *SAMDC1* genes reverted *gfp* silencing in *N. benthamiana* 16c (Zhang et al., 2011).

#### Suppression of TGS by Inactivating Adenosine Kinase and Stabilizing SAMDC1

Adenosine kinase phosphorylation of adenosine is a prerequisite for sustaining cellular SAM levels. Thus, AC2-mediated interaction and inactivation of ADK invoked the possibility of methylation-mediated repression of viral genome as a possible host defense mechanism against the virus. The *Arabidopsis* mutants *adk*, *cmt3*, *drm1/2*, *drb3*, *clsy1*, *pol IV*, and *pol V* (mutants of RNA-directed DNA methylation components) were hypersensitive to viral infection. Cytosine methylation level of the viral genome was significantly reduced in the hypersusceptible mutant plants (Raja et al., 2008, 2014; Jackel et al., 2016). Infection of *Arabidopsis* with a BCTV mutant lacking *C2* (BCTV C2^−^) resulted in a host recovery phenotype. The viral DNA in the recovered tissue was hypermethylated, suggesting methylation of the viral genome to be the cause of host recovery. Interestingly, *ago4*, *dcl3*, *drb3*, and *pol V* mutant *Arabidopsis* plants did not recover when infected with BCTV C2^−^ (Raja et al., 2008, 2014; Jackel et al., 2016), whereas *pol IV* and *clsy1* mutants displayed a delayed recovery phenotype (Jackel et al., 2016). This finding underlined the requirement of AGO4, DCL3, DRB3, Pol IV, and Pol V for methylation of viral genome and resultant host recovery. These results confirmed that methylation of viral genome has been evolved as an epigenetic defense mechanism against geminiviruses (Raja et al., 2008, 2014; Jackel et al., 2016; Coursey et al., 2018).

The AC2/C2-mediated suppression of methylation was studied in the *N. benthamiana* (16-TGS) line, in which the 35S promoter-driven *gfp* transgene is transcriptionally silenced. The silencing of the 16-TGS plant was suppressed by wild-type *AC2* and *AC2*-∆AD of TGMV and CaLCuV and also by BCTV *C2* when expressed from PVX vectors (Buchmann et al., 2009). Knocking down of SAHH and ADK expression using a *Tobacco rattle virus* VIGS vector reversed *gfp* silencing in 16-TGS. These observations further implied that reversal of silencing mediated by inhibition of methyl cycle is one of the prominent mechanisms evolved by viruses to suppress silencing (Buchmann et al., 2009). Infection of *N. benthamiana* 16-TGS with TGMV, CaLCuV, and BCTV restored GFP fluorescence in a manner consistent with the tissue tropism exhibited by the virus. TGMV and CaLCuV infection restored GFP expression in symptomatic vascular and mesophyll cells (Buchmann et al., 2009; Raja et al., 2010). Interestingly, BCTV-mediated suppression *of gfp* silencing was confined to the vascular tissue, which corroborated well with the vascular specificity of the virus. Transgenic expression of *AC2* and *AC2*-∆AD of TGMV, BCTV *C2*, and ds*ADK* under a dexamethasone (dex)-inducible promoter reversed the methylation of transcriptionally silenced loci in *Arabidopsis*. The reversal of methylation by *AC2* and *AC2*-∆AD of TGMV, and BCTV *C2* was found to be locus non-specific. Four independent regions, including one gene-coding region, two transposable elements, and one repetitive DNA region were used as markers to study the effect of *AC2* and *AC2*-∆AD of TGMV, and BCTV *C2* on cytosine methylation. Reversal of cytosine methylation resulted in ectopic expression of TGS-silenced loci namely, a putative F-box gene, a retrotransposon *At*SN1 (SINE element), and *Athila* (LTR element) in all analyzed transgenic plants. The ability of *AC2* and *AC2*-∆AD of TGMV and BCTV *C2* to suppress TGS indicates that the suppression is activation domain-independent. TGS in the CACTA-like transposon was reversed only upon wild-type *AC2* induction. This invoked the necessity of transcriptional activation domain for mediating suppression of the CACTA TGS-loci. Thus, *AC2* transgenic plants reduced cytosine methylation of a wide spectrum of genomic regions and reversed methylation of the TGS loci in *Arabidopsis* by non-specifically inhibiting cellular transmethylation reactions (Buchmann et al., 2009). Although, methylation extension assays confirmed a decrease in 5-methylcytosine methylation of a wide range of genomic regions in *AC2* and *C2* transgenic lines (Buchmann et al., 2009), a whole-genome bisulfite sequencing would provide data on AC2 or C2-mediated reversal of genome-wide cytosine methylation at single-nucleotide resolution.

The attenuation of SAMDC1 degradation by BSCTV C2 may also result in suppression of TGS. SAMDC1 stabilization resulted in an increase in dcSAM/SAM ratio, which in turn reduced the host *de novo* methylation. BSCTV-C2 mutant infection resulted in enhanced DNA methylation of the viral genome (Zhang et al., 2011). In an interesting assay, an *FWA* genomic fragment containing tandem repeats in its promoter region was used to study the role of BSCTV C2 on *de novo* methylation. Methylation of the *FWA* promoter and consequent silencing of the *FWA* gene is essential for normal flowering. The absence of *de novo* methylation of *FWA* transgene in a MTase double mutant *Arabidopsis* plant (*drm1*/*drm2*) caused a delay in flowering (Cao and Jacobsen, 2002). Similar late flowering phenotype was observed when the *FWA* gene was transformed into C2-expressing transgenic lines (Zhang et al., 2011). This suggested a role for BSCTV-C2 in interfering with the *de novo* methylation of the *FWA* transgene.

#### Developmental Stage-Specific Silencing Suppression

CaLCuV AC2 with the transcription activation domain deletion, and BCTV C2 reversed PTGS and TGS in the vegetative phase of plants indicating a transcription-independent mechanism of silencing suppression. Interestingly, only CaLCuV AC2 but not BCTV C2 could reverse PTGS and TGS upon onset of flowering, indicating that a transcription-dependent activity is required during the reproductive transition. A third suppression mechanism was demonstrated in which CaLCuV AC2_1–114_ lacking the transcription activation domain but not BCTV C2 effectively reversed TGS in reproductive plants (Jackel et al., 2015). Although TGS reversal was observed only in the vegetative phase of plants (plants with prolonged vegetative growth), reduced ADK activity was observed in both vegetative and reproductive plants upon BCTV infection or by silencing of ADK suggesting that TGS and ADK inhibition are uncoupled in reproductive *N. benthamiana* plants. In contrast, SAHH silencing resulted in reversal of TGS in both vegetative and reproductive plants. Thus, a new mechanism of TGS reversal that is independent of both transcription activation and ADK inactivation was observed in reproductive *N. benthamiana* plants expressing AC2_1–114_.

#### Suppression of TGS by Inhibiting KYP

Interestingly, TGMV and CaLCuV AC2 were shown to inhibit the enzymatic activity of KYP by binding to the catalytic domain and thereby decreasing CHH methylation in gene-rich regions (Castillo-González et al., 2015). Over-expression of KYP enriched H3K9me2 mark of viral chromatin, leading to formation of viral heterochromatin. As a counter-defense strategy, inhibition of KYP activity by TGMV and CaLCuV AC2 protein restored the euchromatic status of the minichromosome which allowed active replication and transcription of viral genes and suppression of the host defense mechanism (Figure 4F). While *kyp* mutant was hypersusceptible to CaLCuV infection and accumulated significantly higher viral titers when compared to wild-type, over-expression of the *KYP* transgene in the *kyp* mutant reduced the disease severity significantly.

The necessity of functional AC2 for ssDNA accumulation (Hayes and Buck, 1989) and the requirement of AC2-mediated transactivation of the *CP* and *NSP* genes for systemic infection (Sunter and Bisaro, 1992) is well-known. CaLCuV AC2^−^ strain was engineered by introducing a premature stop codon and was used to infect wild-type and *kyp* mutant plants. While CaLCuV lacking functional AC2 did not show any symptom or systemic accumulation of the mutant virus in wild-type plants, the *kyp* mutants showed low level of sustained systemic infection of the mutant CaLCuV. Although, the mutant viral titer was very low in *kyp* mutants, this exciting study confirmed the role of *KYP* in inhibiting viral replication. Whole genome bisulfite sequencing revealed that AC2 inhibited KYP-dependent CHH methylation. Interaction of AC2 with KYP blocks its methylation activity and relaxes the viral chromatin and facilitates viral replication (Figure 4F; Ré and Manavella, 2015).

#### Suppression of TGS by Ectopic Expression of VIM5

A recent work by Chen et al. (2020) revealed a unique mechanism in which BSCTV-encoded C2 recruits a host imprinted gene VIM5 to evade host silencing. While over-expressing BSCTV *Rep* transgene that also contained the *C2*-*C3* promoter sequence and C2 N-terminal sequence (C2_N_), the authors serendipitously observed accumulation of the *C2_N_* transcript in addition to the *Rep* transcript in one of the transgenic *Arabidopsis* lines. Additional transcription from the *C2*-*C3* promoter was correlated with hypomethylation of the *C2*-*C3* promoter. Ectopic vegetative transcription of the endosperm-imprinted E3 ubiquitin ligase-encoding gene *VARIANT IN METHYLATION5* (*VIM5*) was observed only in the *C2_N_* transcript-expressing transgenic line, when compared to wild-type *Arabidopsis*. The role of VIM5 in hypomethylation was substantiated by over-expressing *VIM5* in another Rep transgenic line that did not originally accumulate the *C2_N_* transcript; Over-expression of *VIM5* triggered accumulation of the *C2_N_* transcript which correlated with decreased DNA methylation at the transgenic *C2-C3* promoter. Infection with BSCTV resulted in transient expression of the host endosperm-imprinted E3 ubiquitin ligase-encoding gene *VIM5* in rosette leaf tissues. BSCTV infection of *Arabidopsis vim5* mutants resulted in delayed accumulation of viral early gene transcripts *C2* and *C3*, thus confirming the role of VIM5 in contributing to the early expression of C2 and C3 from the viral genome. VIM5 was shown to interact with host CG MTase MET1 and the CHG MTase CMT3 and promote 26S proteasomal degradation of MET1 and CMT3. MTase mutant plants *met1* and *cmt3* displayed reduced viral methylation at the *C2*-*C3* promoter. Similarly, infection of a modified BSCTV with substitutions at CG and CHG sites in the *C2*-*C3* promoter showed higher viral accumulation in *vim5* mutant when compared with the unmodified BSCTV. Thus, early expression of BSCTV silencing suppressor C2 and replication enhancer protein C3 immediately after the expression of Rep, by activating an imprinted E3 ubiquitin-ligase gene, is a strategy evolved by the virus to inhibit viral DNA methylation and establish disease (Chen et al., 2020).

#### Suppression of PTGS by Inhibiting RDR6 and AGO1

Silencing suppression activity of MYMIV AC2 was demonstrated *in gfp* silenced *Nicotiana xanthi* plants (Karjee et al., 2008; Rahman et al., 2012). The activation domain mutant of MYMIV-AC2 possessed the suppression activity. A two-pronged mechanism involving physical interaction of AC2 with RDR6 and AGO1 was shown as the basis of PTGS suppression of MYMIV AC2 (Figure 3A). AC2-mediated inhibition of RNA-dependent RNA polymerase activity and slicing activity of AGO1 protein resulted in reduced siRNA accumulation (Figure 3A; Kumar et al., 2015). An *in planta* assay using invert repeat of RDR6 to reverse *gfp* silencing only partially mimicked the suppression activity of AC2. The possibility of RDR6 paralogs partially compensating for the absence of RDR6 cannot be discounted. Future studies involving *rdr6* and *ago1* mutant plants and testing them for enhanced susceptibility would reveal any redundant function of paralogs.

### Suppression of Defense Mechanism by Inactivating Cellular Regulatory Genes

#### Inactivation of SNF1 Kinase

AC2 of TGMV and C2 of BCTV have been shown to interact with SNF1-related kinase (SnRK1), a serine-threonine kinase, and inactivate it (Hao et al., 2003). SnRK1 has been implicated as a key regulator of cellular stress response including innate defense mechanism. AC2-SnRK1 interaction alters the cellular stress metabolism and causes a novel enhanced susceptibility (Hao et al., 2003). Although SNF1 inactivation does not involve suppression of silencing, interaction of AC2 with ADK reduces the cellular AMP levels (Figure 4D; Wang et al., 2003). AMP acts as an activator of SNF1 (Figure 4G), suggesting a dual strategy evolved by the virus to attenuate the cellular metabolism by inactivating SNF1 by direct interaction and indirectly by reducing cellular AMP levels (Figure 4G). *Arabidopsis* SnRK1 was shown to phosphorylate the serine residue at 109 (S^109^) of CaLCuV AC2 protein. A phosphomimic mutation of the S^109^ reduced viral DNA accumulation and delayed symptom appearance in *Arabidopsis*, thus revealing phosphorylation of viral protein as a host defense mechanism against an invading virus (Shen et al., 2014). Guerrero et al. (2020) report that AC2 of all old world begomoviruses and cutoviruses C2 lack the S^109^ residue. While majority of the new world begomovirus AC2s possess the conserved SnRK1 phosphorylation site, some new world begomoviruses including TGMV AC2 lack the S^109^ residue and hence are not phosphorylated. Instead, TGMV AC2 is shown to inhibit the kinase activity of SnRK1 (Hao et al., 2003).

#### Elevation of Cellular Cytokinin Levels

Baliji et al. (2010) demonstrated a novel consequence of ADK inhibition by AC2 of TGMV and C2 of SCTV. ADK has a role in maintaining the cellular cytokinin level. ADK-mediated phosphorylation of cytokinin converts the bioactive form of cytokinin to a less bioactive form (Figure 4H). Silencing of ADK in *Arabidopsis* increased the cellular cytokinin levels, substantiated by increased activity of a cytokinin-responsive promoter. Over-expression of CaLCuV AC2 and SCTV C2 in *Arabidopsis* resulted in increased expression of endogenous cytokinin-responsive promoters. Thus, geminivirus AC2, by inactivating ADK, increased the cellular cytokinin levels. An enhanced cytokinin level is a prerequisite for cell cycle progression and to maintain an active state of replication. Increased cytokinin resulted in an enhanced susceptibility phenotype. Interestingly, cytokinin is known to negatively regulate the expression of SULTR1;2, a high affinity sulfate transporter in *Arabidopsis* roots (Maruyama-Nakashita et al., 2004). Cytokinin binding to the receptor cytokinin response 1/wooden leg/*Arabidopsis* histidine kinase 4 (CRE1/WOL/AHK) is the cue for negative regulation of sulfur assimilation, while *cre1* mutant is insensitive to cytokinin. In *sultr1;2* mutant, SULTR1;1 was downregulated by cytokinin. Downregulation of sulfate transporters by cytokinin correlated with the decrease in sulfate uptake. Another study by Ohkama et al. (2002) showed that exogenous application of cytokinins upregulated sulfur responsive genes APS reductase 1 (*APR1*) and *SULTR1;2* through a pathway independent of sulfur starvation; likely through increasing sucrose concentration which is known to upregulate *APR1* gene (Kopriva et al., 1999). Exogenous application of cysteine and glutathione (GSH) resulted in downregulation of ATP sulfurylase, APS reductase, and sulfate transporter (Vauclare et al., 2002).

TYLCSV-*C2* transgenic plants repressed the expression of three genes involved in sulfur assimilation namely ATP sulfurylase 3 (*APS3*), APS reductase 1 (*APR1*), and APS reductase 3 (*APR3*) and also accumulated reduced sulfur (Lozano-Durán et al., 2012). Adequate sulfate supply in tobacco plant resulted in a suppressed and delayed *Tobacco mosaic virus* (TMV) symptom development through a phenomenon named sulfur-induced resistance (SIR) or sulfur-enhanced defense (SED; Höller et al., 2010), thus revealing a role for sulfur in plant defense. Exogenous treatment of the TYLCSV-*C2* plants with methyl jasmonate (MeJA) reversed the repression of sulfur assimilation genes (Lozano-Durán et al., 2012). TYLCSV-*C2* transgenic plants over-accumulated cysteine and glutathione. The authors could not explain the reason behind the over-accumulation of cysteine and glutathione when the sulfur assimilation genes were repressed. Lozano-Durán et al. (2012) proposed that the C2 protein might suppress SIR/SED by suppressing jasmonate signaling pathway. However, it would be interesting to study in future if TYLCSV-*C2* plants had increased cellular cytokinin levels similar to TGMV-*AC2*, CaLCuV-*AC2*, and SCTV-*C2* plants. Also, it is relevant to study if upregulation of sulfur assimilation genes by cytokinin is initially through sucrose-dependent pathway. It is also important to study if the reduction observed in the expression of sulfur assimilation genes is because of the accumulation of cysteine and glutathione in TYLCSV-*C2* transgenic plants. It would be informative to understand if the sulfate reduction in C2 transgenic plants might be because of the negative regulation of sulfate transport pathway by cytokinin binding to CRE1 receptor. Further studies are necessary to understand cross-talk between cytokinin, jasmonate, and sulfur assimilation pathways.

#### Inhibition of Jasmonate Signaling Pathway

COP9 signalosome (CSN), a highly conserved protein complex with eight subunits that resembles the 19S lid of the 26S proteasome (Dohmann et al., 2008), regulates the activity of E3 ligases. The CSN complex comprises eight subunits, named CSN1–CSN8, where CSN5 is the only catalytic subunit. TYLCSV C2 interacts with CSN5 and interferes with the cellular ubiquitination machinery (Lozano-Durán et al., 2011). Ubiquitination occurs through a cascade of enzymatic reactions namely ubiquitin activation by E1, conjugation by E2, and ligation by E3. E3 ligase comprises the multisubunit Cullin RING Ligases (CRLs). Among CRLs, cullin 1-based group or SCF (for Skp1/Cullin1/F-box), is comprised of four proteins, cullin 1 (CUL1), S-phase kinase-associated protein (SKP1/ASK), the RING subunit RBX1 (RING box 1) and an F-box substrate binding protein. The CRL activity is regulated by covalent attachment and removal of ubiquitin-like protein RUB (related to ubiquitin; Figure 4I). CSN5 is associated with the derubylation activity (Gusmaroli et al., 2007). CSN is a multisubunit isopeptidase which removes the RUB moiety from CRLs to function *in vivo*. C2 of TYLCSV interacts with CSN5 and inhibits the derubylation of CRL (Figure 4I). Rubylated CUL1 alters several SCF-dependent hormonal processes and also suppresses jasmonate responses in *C2* transgenic plants. Jasmonate signaling has been implicated in defense response and suppression of jasmonate response resulted in enhanced susceptibility phenotype, which was reverted upon exogenous treatment of methyl jasmonate (MeJA). TYLCV C2 and BCTV C2 were also shown to interact with CSN5 suggesting that CSN5-C2 interaction is a conserved function in geminiviruses (Lozano-Durán et al., 2011).

Jasmonate receptor SCF complex is also the receptor for the bacterial toxin coronatine, which is secreted by the plant pathogenic *Pseudomonas syringae* pv. tomato DC3000 (Pto DC3000). Coronatine application on plants facilitates stomatal opening and increases infection (Melotto et al., 2006; Geng et al., 2014). The hindrance of SCF complex by TYLCV C2 (Lozano-Durán et al., 2011) makes TYLCV/TYLCSV C2 transgenic plants less sensitive to coronatine, as evidenced by reduced Pto DC3000 bacterial growth (Rosas-Díaz et al., 2016). The mutant COR^−^ strain and wild-type strain had similar bacterial growth in C2 transgenic plants (Rosas-Díaz et al., 2016) confirming the role of C2 in altering SCF function (Lozano-Durán et al., 2011).

Jasmonic acid, which is involved in biotic and abiotic stress responses, is also known to induce the production of secondary metabolites including alkaloids, anthocyanins, and terpenoid compounds (Devoto et al., 2005). Whitefly-infested plants showed increased terpenoid production which was decreased in virus-infected plants (Luan et al., 2013). TYLCV-infected tobacco plants manifested increased survival and fecundity of whiteflies (Li et al., 2019). Terpene synthesis genes were downregulated in TYLCV and TYLCV-whitefly co-infected plants compared to whitefly-infested plants. TYLCV *C2* over-expression lines had decreased expression of terpene synthesis genes including MYC2, a transcription factor. MYC2 is under the regulatory control of JAZ1 protein and ubiquitination-mediated degradation of JAZ1 protein is the switch of MYC2 activation (Niu et al., 2011). TYLCV and *Papaya leaf curl China virus* (PaLCuCNV) C2 were shown to interact with RPS27A, a fusion protein consisting of ubiquitin at the N terminus and ribosomal protein S27a at the C terminus. C2 interaction was with the ubiquitin moiety of RPS27A. Li et al. (2019) demonstrated a novel suppression mechanism in which TYLCV C2 competitively bound to ubiquitin, which resulted in decreased JAZ1 protein ubiquitination (Figure 4J). Consequently, the MYC2 bound to JAZ1 is stabilized and interferes with the ability of MYC2 to induce the expression of downstream defense genes.

#### Suppression by Inhibiting Cell Cycle Regulator

*Arabidopsis* PEAPOD2 transcription factor (TIFY4B) was shown to interact with *AC2* and promoter sequences of TGMV and CaLCuV *CP* (Lacatus and Sunter, 2009) and TGMV *NSP* (Berger and Sunter, 2013). TIFY4B is known to limit cell proliferation in leaf epidermis and vascular tissues (White, 2006), which is substantiated by increased TIFY4B expression in callus tissue and during inflorescence emergence (Vanholme et al., 2007). TIFY4B has three conserved domains: PPD, TIFY, and CCT_2. While TIFY4B was shown to localize to nucleus (Lacatus and Sunter, 2009), mutant versions of TIFY4B including an 84–150-amino acid version were localized in the cytoplasm (Chung and Sunter, 2014). TGMV and CaLCuV AC2 interaction with the mutant TIFY4B altered the localization to nucleus. Increased TIFY4B expression was observed upon viral infection while the geminivirus infection is expected to downregulate a repressor of cell cycle progression. Over-expression of TIFY4B resulted in increased mean latent period and reduced CP expression suggesting a role of TIFYB in antiviral defense, wherein TIFYB inhibits cell proliferation, and therefore, viral replication. Chung and Sunter (2014) proposed a suppression mechanism in which AC2 sequesters TIFY4B and inhibits its role in cell cycle regulation, thereby creating a conducive environment for viral replication.

## Summary

This review highlights the different targets and mechanisms evolved by geminivirus AC2/C2 to counter PTGS and TGS. Recent studies on cellular proteins that are targets of AC2/C2 have opened a third defense mechanism which does not involve PTGS and TGS (Table 1). Many studies show how a single suppressor protein like TGMV AC2 could deploy multiple targets. TGMV AC2 targets rgsCaM and ADK to suppress PTGS, targets ADK, and KYP to suppress TGS and inactivates SnRK1 and TIFY4B to inhibit the cellular defense response (Table 1). It is likely that silencing suppression is not limited to targeting of one viral protein and a host protein but rather involves a concerted effect on multiple host proteins as manifested in TGMV AC2. It would be interesting to see if other viral suppressor proteins also act on multiple targets. Most of our current knowledge regarding suppressor proteins and their targets is from curtoviruses and begomoviruses. Positional homologs of AC2/C2 are observed in other geminiviruses namely *Eragovirus*, *Topocuvirus*, and *Turncurtovirus*; however, they are not known to encode functional AC2/C2. Advancing our knowledge of suppressor proteins in other genera is essential. For example, *Grapevine red blotch virus* (GRBV), the type member of *Grablovirus*, is known to be a serious threat to the Californian wine industry. Understanding the GRBV suppressor proteins and their plant targets can present cogent strategies for mitigating this threat to a multibillion-dollar industry. Most of our current knowledge on silencing and silencing suppression is from dicot plants *Arabidopsis* and *N. benthamiana*. The genus *Mastrevirus* are monocot-infecting geminiviruses with 41 species. The only silencing suppressor protein identified till date in *Mastrevirus* is the Rep protein from *Wheat dwarf virus* which binds to ss- and ds-siRNA (Wang et al., 2014). It would be interesting to see if DCL5 and AGO18, the unique silencing machinery components of monocots, are targets of *Mastrevirus*.

**Table 1:** Geminivirus AC2/C2-plant protein interactions and associated functions.

Virus	Suppressor	Suppressing PTGS	Suppressing TGS	Cellular pathways
MYMV	AC2	Upregulates host suppressor protein WEL1 (Trinks et al., 2005)		
TGMV BCTV	AC2 C2	Inactivates adenosine kinase (Wang et al., 2003, 2005)	Inactivates adenosine kinase (Buchmann et al., 2009)	Inactivates a serine-threonine kinase SnRK1 (Hao et al., 2003)
BSCTV	C2	Stabilizes S-adenosyl methionine decarboxylase1 (SAMDC1; Zhang et al., 2011)	Activates VIM5, an endosperm-imprinted E3 ubiquitin-ligase gene (Chen et al., 2020)	
TGMV CaLCuV	AC2 AC2	Upregulates rgsCaM (Chung et al., 2014)	Inhibits histone methyltransferase KYP (Castillo-González et al., 2015)	Sequesters PEAPOD2 transcription factor (TIFY4B) and inhibits cell cycle regulation (Lacatus and Sunter, 2009; Chung and Sunter, 2014)
TGMV SCTV	AC2 C2			Elevation of cellular cytokinin levels (Baliji et al., 2010)
TYLCSV TYLCV	C2 C2			Interacts with CSN5 and inhibits jasmonate signaling (Lozano-Durán et al., 2011; Rosas-Díaz et al., 2016) Represses sulfur assimilation genes (Lozano-Durán et al., 2012)
ICMV-SG	AC2		Upregulation of RAV2, transcription repressor and repression of H3K9 histone methyltransferase KRYPTONITE (KYP; Sun et al., 2015)	
MYMIV	AC2	Interacts with RDR6 and AGO1(Kumar et al., 2015)		
TYLCV	C2			Downregulates terpene synthesis (Luan et al., 2013).
TYLCV PaLCuCNV	C2 C2			Interaction with the ubiquitin moiety of RPS27A resulting in decreased JAZ1 degradation (Li et al., 2019)

MYMV, *Mungbean yellow mosaic virus*; TGMV, *Tomato golden mosaic virus*; BCTV, *Beet curly top virus*; BSCTV, *Beet severe curly top virus*; CaLCuV, *Cabbage leaf curl virus*; SCTV, *Spinach curly top virus*; TYLCSV, *Tomato yellow leaf curl Sardina virus*; TYLCV, *Tomato yellow leaf curl virus*; MYMIV, *Mungbean yellow mosaic India virus*; ICMV-SG, *Indian cassava mosaic virus*-Singapore; and PaLCuCNV, *Papaya leaf curl China virus*.

## Future Directions

Future research in AC2 suppressor protein should be focused on addressing the question of how the small AC2/AL2 protein (15 kDa) has evolved an ability to interact with so many different targets and make an impact on viral pathogenesis and plant metabolism. It would be interesting to know which of the studied target/targets is relevant in viral pathogenesis. Editing of the target genes in plants and study of viral pathogenesis and RNA silencing in the mutant plants infected with viruses will help in a more thorough evaluation of the AC2-interacting plant proteins. *Abutilon mosaic virus* (AbMV) AC2 was shown to act as a brake in geminivirus replication and was reported to enhance PTGS rather than suppress it (Krenz et al., 2015). It would be interesting to study if any other geminiviral AC2/C2 function as a facilitator of silencing. Besides the uniqueness of AbMV AC2 acting as a PTGS enhancer, a greater challenge is to understand how AbMV AC2 evolved as an enhancer. Does this confer any advantage to the virus or did the host evolve to inhibit viral replication or symptom development?

One major focus of future study should be in exploiting AC2 silencing suppressor function for biotechnological applications. The toxic effect of AC2, when expressed as a transgene, has been reported by several groups. This has limited the use of geminivirus AC2 in transgenic technology. Mutations of functional domains have been shown to alleviate the toxic effect (Rajeswaran et al., 2007). A study to precisely identify the amino acid residue/residues that contribute to the toxic effect of the 135 aa AC2 protein is highly desirable. DNA viral vectors containing only the replication origin and with Rep gene provided in *cis* or in *trans* have found application in biopharming (Rybicki and Martin, 2014). *Bean yellow dwarf virus* (BeYDV), a mild dicot infecting *Mastrevirus*, has been deconstructed and used widely as a replicon vector (Regnard et al., 2010). Huang et al. (2010) demonstrated high level expression of Ebola virus glycoprotein GP1 (6D8) recombinant monoclonal antibodies in *N. benthamiana* leaves by co-expressing the transgene in BeYDV replicon along with Rep and *Tomato bushy stunt virus* (TBSV) p19, a ds siRNA binding suppressor protein. Earlier work by Lacombe et al. (2018) has demonstrated that a combination of P19, P0, and P1 suppressors, that act at distinct steps of the RNA silencing pathway, allowed the highest ectopic protein expression. If the toxic effect of geminiviral AC2 can be negated without compromising on the silencing suppression function, *AC2*, which is known to target multiple steps of gene silencing, could find extensive use for over-expressing recombinant proteins in plants and plant cell cultures.

Both RNA and DNA viruses are widely used in Virus-Induced Gene Silencing (VIGS). The ease with which DNA viruses can be manipulated, the fact that geminiviral *CP* can be replaced with the gene of interest without affecting the systemic movement of virus (only in certain viruses), the recombinant vector derived from replacing *CP* is not vector transmissible and the broad host range of DNA viral vectors makes DNA viruses a good choice for VIGS over RNA viruses (Robertson, 2004). While several studies have shown the application of geminivirus as VIGS vector (Yang et al., 2017), the presence of suppressor proteins including AC2/C2 was shown to antagonize the silencing effect caused by the VIGS vector. The silencing efficiency is depleted as the suppressor protein accumulates in the plants. A null mutant in MYMIV-AC2, when used as VIGS vector, increased the silencing efficiency (Pandey et al., 2009). However, the mutation in the AC2 ORF reduced MYMIV replication efficiency by about 25%. A null mutation in CaLCuV AC2 abolished the viral replication (Castillo-González et al., 2015). Hence, future studies should investigate the possibility of mutating the AC2/C2 to mute the suppression function alone without compromising on the replication efficiency of the VIGS vector. Geminivirus VIGS vectors will help in performing functional genomics in a wide range of plants.

Baltes et al. (2014) demonstrated genome engineering using the BeYDV-based replicon system. Desired DNA changes were made when TALENs and CRISPR/Cas9 system were delivered using the BeYDV-based replicon system. A recent study by Mao et al. (2018) showed improved gene-editing efficiency by silencing AGO1 or by co-expressing TBSV p19 protein as part of CRISPR/Cas9 cassette. The challenge of gene editing technology is in identifying the edited events. Mao et al. (2018) established a suave means to phenotypically identify the edited events in the T_1_ generation and T-DNA segregated events in T_2_ generation. By co-expressing p19 as part of the CRISPR/Cas9 cassette, the authors could group the T_1_ plants based on the severity of p19-induced leaf phenotype alteration. T_1_ plants displaying severe phenotype associated with p19 over-expression were selected as edited lines. T_2_ seedlings from the gene edited T_1_ events were again grouped based on the leaf phenotype. Segregated plants with wild-type leaf phenotype were then identified as gene-edited plants from which the T-DNA was segregated out. The geminiviral suppressor protein AC2 can be co-expressed to increase the genome editing efficiency. Interestingly, AC2 is more attractive than TBSV p19 for the following reasons: (1) AC2 over-expression is known to cause pronounced phenotype in leaf (Siddiqui et al., 2008; Castillo-González et al., 2015). (2) TGMV AC2 over-expression resulted in early flowering (Castillo-González et al., 2015). (3) AC2 over-expression is likely to result in genome-wide hypomethylation (Castillo-González et al., 2015), and hence the tightly regulated genes involved in meiotic recombination are also likely to be upregulated. (4) MYMV AC2 and ACMV AC2, when transiently expressed in *Arabidopsis* protoplasts, upregulated the expression of the meiotic recombination protein AtDMC1 (Trinks et al., 2005; Da Ines et al., 2013). Thus, future studies would pave way to the evolution of AC2 from a core viral protein to a potent molecular tool with myriad applications.

## Author Contributions

SS: conceptualization and writing-original draft preparation. KV: conceptualization, reviewing, and editing. Both authors contributed to the article and approved the submitted version.

### Conflict of Interest

The authors declare that the research was conducted in the absence of any commercial or financial relationships that could be construed as a potential conflict of interest.
